# Rare damaging variants in DNA repair and cell cycle pathways are associated with hippocampal and cognitive dysfunction: a combined genetic imaging study in first-episode treatment-naive patients with schizophrenia

**DOI:** 10.1038/tp.2016.291

**Published:** 2017-02-14

**Authors:** Z Yang, M Li, X Hu, B Xiang, W Deng, Q Wang, Y Wang, L Zhao, X Ma, P C Sham, G Northoff, T Li

**Affiliations:** 1 The State Key Laboratory of Biotherapy, Psychiatric Laboratory, West China Hospital, Sichuan University, Chengdu, Sichuan, China; 2 The Mental Health Center, West China Hospital, Sichuan University, Chengdu, Sichuan, China; 3 Biobank, West China Hospital, Sichuan University, Chengdu, Sichuan, China; 4 Centre for Genomic Sciences and Department of Psychiatry, University of Hong Kong, Pokfulam, China; 5 Institute of Mental Health Research, University of Ottawa, Ottawa, ON, Canada

## Abstract

Schizophrenia is a complex neurodevelopmental disorder where changes in both hippocampus and memory-related cognitive functions are central. However, the exact relationship between neurodevelopmental-genetic factors and hippocampal-cognitive dysfunction remains unclear. The general aim of our study is to link the occurrence of rare damaging mutations involved in susceptibility gene pathways to the structure and function of hippocampus in order to define genetically and phenotypically based subgroups in schizophrenia. In the present study, by analyzing the exome sequencing and magnetic resonance imaging data in 94 first-episode treatment-naive schizophrenia patients and 134 normal controls, we identified that a cluster of rare damaging variants (RDVs) enriched in DNA repair and cell cycle pathways was present only in a subgroup including 39 schizophrenic patients. Furthermore, we found that schizophrenic patients with this RDVs show increased resting-state functional connectivity (rsFC) between left hippocampus (especially for left dentate gyrus) and left inferior parietal cortex, as well as decreased rsFC between left hippocampus and cerebellum. Moreover, abnormal rsFC was related to the deficits of spatial working memory (SWM; that is known to recruit the hippocampus) in patients with the RDVs. Taken together, our data demonstrate for the first time, to our knowledge, that damaging rare variants of genes in DNA repair and cell cycle pathways are associated with aberrant hippocampal rsFC, which was further relative to cognitive deficits in first-episode treatment-naive schizophrenia. Therefore, our data provide some evidence for the occurrence of phenotypic alterations in hippocampal and SWM function in a genetically defined subgroup of schizophrenia.

## Introduction

Schizophrenia, which affects ~1% of the population, is a devastating mental disorder characterized by a range of symptoms including abnormal perception, thinking, cognitive, emotion and social behavior and so on. It is widely accepted that a complex interplay of genetic and environmental factors contributes to the etiology of schizophrenia,^1^ which therefore is regarded a neurodevelopmental disorder.^2, 3^ During the last decades, researchers have made significant strides in teasing apart schizophrenia's convoluted genetic vulnerabilities. These included initial epidemiology studies within family, twin and adoption,^4, 5^ and then linkage studies mainly using microsatellite markers^6^ to candidate genes and genome-wide association studies.^1^

Recently, genome-wide association studies identified that some common variants with small to moderate effects as well as rare but gene-disrupting copy number variants are significantly associated with schizophrenia.^1, 2, 3, 7^ Studies using the whole-exome sequencing technologies have also revealed that rare damaging nonsense and loss-of-function variants and/or *de novo* variants conferred higher risk to schizophrenia. However, to validate these variants in population remains challenging because of the low frequency and effect sizes.^8, 9, 10, 11, 12, 13^ Converging evidence from studies of common and rare variants identified broad classes of genes and pathways involved in calcium ion channel, synaptic plasticity and neurodevelopment processes,^9, 12, 13, 14^ chromatin remodeling and synaptic network.^12, 15^ Although these results show significant leads for identifying susceptibility genes of schizophrenia, the exact and specific genetic factors contributing to the abnormal neurodevelopment processes in schizophrenia remain inconclusive.

A key brain region in schizophrenia is the hippocampus^16, 17^ where reduced volume in especially left (rather than right) hippocampus^18^ as well as resting-state functional connectivity (rsFC) differences to other regions^19, 20^ have been observed. This corresponds to the observation of neuropsychological deficits, especially dysfunctional spatial working memory (SWM) and logical memory in patients with schizophrenia.^21, 22, 23^ However, the exact linkage between hippocampus and memory deficits on the one hand and underlying genetic-neurodevelopmental changes on the other hand remain unclear.

The general aim of our study was to conduct multilevel genetic imaging cognitive investigation in a unique large sample of first-episode treatment-naive schizophrenic patients. For that purpose, we combined genetic analysis of neurodevelopmentally related genes with structural and functional imaging of the hippocampus and related cognitive measures. Specifically, genetic investigation in schizophrenic (and healthy) subjects served to determine specific genetic neurodevelopmentally relevant subtypes, which were then further characterized phenotypically using structural and functional hippocampal imaging as well as related cognitive, that is, memory measures.

The first specific aim was to unravel unique rare damaging variants (RDVs) in a unique sample of first-episode treatment-naive schizophrenia patients relative to healthy controls by using whole-exome sequencing approach, and then apply Gene Ontology (GO) enrichment strategy to identify gene pathways. Consequently, we focused on the RDVs in DNA repair and cell cycle pathways for the following reasons. First, the majority of these genes in DNA repair and cell cycle pathways are most highly expressed in various brain regions, such as hippocampus;^8, 9, 10, 11, 12, 13^ previous studies suggested indeed that genes involved in the regulation of cell cycle and DNA repair significantly influence the hippocampus function in schizophrenia.^24, 25, 26, 27, 28^ However, how the expression profile of these genes in cell cycle and DNA repair pathways map spatially and temporally during the critical neurodevelopmental stage in specifically the hippocampus in schizophrenia remains unclear.^8, 9, 10, 11, 12, 13^ We therefore hypothesized that we could distinguish two genetic subtypes in schizophrenia, one with RDVs and one without RDVs, as related to hippocampal developmental function.

On the basis of these considerations, the second specific aim consisted of measuring especially rsFC (as controlled for by structural volume) of the hippocampus in the same patients using brain magnetic resonance imaging (MRI), and to explore the relationship among genes, rsFC of the hippocampus and related neurocognitive functions. We hypothesized different hippocampal rsFC patterns in those schizophrenic patients with RDVs when compared with the ones without RDVs. To further underline hippocampal involvement, we included cognitive measures like spatial and logical memory that have been shown to be associated with specifically hippocampal function.^29^ We hypothesized that schizophrenic patients with RDVs may have different memory-related cognitive functions relative to schizophrenic patients without RDVs.

There are several unique advantages in the present study. First, this is the first investigation, to our knowledge, to study the contribution of RDVs to the imaging and neurocognitive phenotype of schizophrenia. Second, we recruited first-episode treatment-naive patients with schizophrenia to rule out the confounding factors of chronicity of the illness and treatment effects on neuroimaging and neurocognitive assessments. Third, extending limits on the power to identifying specific loci responsible for the disorder,^30, 31^ we improve the definition of the phenotype and/or reducing the phenotypic complexity of schizophrenia for genetic studies by combining the latter with cognitive (for example, neuropsychological) and neural (for example, neuroimaging measures) that are more closely related to the phenotype.

## Materials and methods

### Samples

There were total 234 participants including 97 first-episode treatment-naive patients with schizophrenia and 137 healthy controls. Patients with schizophrenia were recruited at the Mental Health Centre of the West China Hospital, Sichuan University, China. Healthy volunteers were recruited from the community. The study was approved by the ethical committee in West China Hospital of Sichuan University. All participants were Han Chinese and provided written informed consent for their participation in this study.

### Clinical and memory assessments

All patients were interviewed by a trained psychiatrist using the Structured Clinical Interview for the DSM-IV (SCID).^32^ DSM-IV criteria for schizophrenia were used for diagnosis. Those who were initially diagnosed with schizophrenia from psychosis due to the illness duration (less than 6 months) were followed up for at least 6 months to meet the DSM-IV criteria for schizophrenia. Psychopathology associated with schizophrenia was evaluated using the positive and negative syndrome scale.^33^ Healthy controls were screened with the SCID-P non-patient version for the lifetime absence of psychiatric illnesses. Subjects with the existence of organic brain disorders, alcohol or drug abuse, pregnancy or any severe physical illness, such as brain tumor or epilepsy, were excluded from the study.

Schizophrenic patients and healthy controls completed SWM test in the Cambridge Neuropsychological Test Automated Battery (CANTAB; http://www.cantab.com) and immediate and delayed (30 min) logical memory subtest of the Wechsler Memory Scale,^34^ respectively.

### MRI data acquisition

Overall, 74 healthy controls and 74 patients with schizophrenia underwent structural MRI scans on a Signa 3.0-T scanner (General Electric, Medical Systems, Milwaukee, WI, USA), whereas 65 healthy controls and 55 patients were scanned to obtain brain resting-state functional MRI in the Department of Radiology at West China Hospital. Detailed procedures of scanning are presented in Supplementary Information, sections 7 and 8.

### Sequencing and variant calling

All samples were sequenced using the TruSeq Exome Enrichment Kit (San Diego, CA, USA) optimized for IlluminaHiSeq2000 sequencing. The pipeline of raw data processing and variants calling is present in Supplementary Figure 2, which includes using Burrows-Wheeler Alignment tool^35^ alignment reads to the reference human genome (hg19); Picard tools (http://picard.sourceforge.net/) to collect quality statistics and fix read group problem; GATK^36^ for IN/DEL alignment; Samtools (http://samtools.sourceforge.net/) to filter out low-quality reads; and GATK to perform SNP and INDEL calling. Validation of selected variants was conducted by Sanger sequencing.

### Data analysis

We use PLINK^37^ and KGGSeq (http://statgenpro.psychiatry.hku.hk/limx/kggseq/doc/UserManual.html)^38^ to perform individual and variants' quality control. We use software KGGSeq to integrate databases to annotate the minor allele frequency of variants, including Hapmap and 1000 Genome. The cutoff frequency was set at 0.1% for rare variance. Methods implemented in PLINK/Seq (http://atgu.mgh.harvard.edu/plinkseq/) were employed for single site and gene-based association analysis. After filtering, we performed GO enrichment analysis in 2895 mutations within 2442 genes in cases and 4484 mutations within 3481 genes in controls using GeneMANIA (http://www.genemania.org/).The results of enrichment indicated that two GO (GO: 0006281 and GO:0007049) only present in cases. We identified more mutations in controls (that is, 2895 mutations within 2442 genes in schizophrenic patients and 4484 mutations within 3481 genes in healthy controls) mainly because, in present study, the sample size in controls (134) is bigger than cases (94). The bigger sample size in controls provides the higher chance to identify mutations. The weighted gene coexpression network analysis (WGCNA)^39^ was used for expression profile, all of which was carried out by the WGCNA R package.^40^ All of Brain expression data from different points in life were acquired from a published study (The Human Brain Transcriptome).^41^ Sample size and statistical power were conducted by the statistical software, Exome Power Calculation (http://darth.ssg.uab.edu:8080/epc/).

rsFC of bilateral hippocampus and six hippocampal subregions was calculated by seed-to-voxel analysis using Statistical Parametric Mapping (SPM8, http://www.fil.ion.ucl.ac.uk/spm) and data-processing assistant for resting-state functional MRI from resting-state functional MRI (Supplementary material). The time courses averaged over all voxels of each hippocampus and six hippocampal subregions were extracted. Pearson's correlation coefficients (*r*) between time courses of left/right hippocampus and all other voxels were calculated and transformed to Fisher's *z*-scores to derive rsFC maps. Thus, 67 349 pairs of functional connectivity were calculated between right hippocampus and the other voxels of the brain, whereas 67 370 pairs of functional connectivity were computed between left hippocampus and the other voxels of the brain. Statistical tests on the functional connectivity maps of hippocampus between patients with schizophrenia and healthy controls were performed using analysis of covariance with sex, age and education years, and volume of hippocampus as covariates in SPM8. The significant threshold was set at *P*<0.05, corrected for multiple comparisons based on Monte Carlo simulations. Subsequently, the mean *Z*-value of each cluster with a significant functional connectivity (FC) difference was extracted and were compared by analysis of variance followed by *post hoc* test (least significant difference) in SPSS 18.0, across patients with RDVs, patients without RDVs and healthy controls (SPSS, Chicago, IL, USA), significant level of P values were set at less than 0.05. Analysis of covariance followed by *post hoc* test were applied to test the differences of functional connectivity and memory functions among patients with RDVs, patients without RDVs and healthy controls. Partial correlation analysis was used to analyze the relationship between the functional connectivities of bilateral hippocampus (and six hippocampal subregions) and cognitive, that is, memory functions, with age, sex and education years (and structural hippocampal volume) as covariance (Supplementary material). Furthermore, Fisher's Z-transformation was used to transform the correlation coefficients(r value) to Z-score, and Z-test was used for testing the differences of correlations between groups. Corrections for multiple comparisons were applied as appropriate.

## Results

### Genetic investigation of single nucleotide variants and In/Dels

In the current study, 234 subjects (97 patients with first-episode treatment-naive schizophrenia and 137 healthy controls) were collected for exome sequencing to identify those alleles, genes or gene networks that harbor rare coding variants of moderate or large effect on risk for schizophrenia (Supplementary Information, sections 1). Six samples were removed with low quality along with likely contamination after quality control and variant calling (Supplementary Information, sections 2 and 3). Quality control of all variants was conducted by in-house software KGGSeq.^38^ The final data set comprised 94 schizophrenic cases and 134 controls. Detailed demographic and clinical information, as well as summary for sequencing quality, was shown in Table 1. There were no significant differences in age, sex distribution and years of education (Table 1).

On average, we obtained 7.67 Gb of mappable sequence data per individual after exome enrichment, targeting ~62 Mb from exons and their flanking regions. In all, 99.75% of the reads were properly aligned to the reference genome. Our median read depth is ~45 ×, which is higher than the estimated average depth (33 ×) required for highly accurate downstream heterozygous variant detection. In addition, 88.60% of the captured target exons were covered by high-quality genotype calls at least 10 times to ensure good detection sensitivity.^42^ Technical sequencing metrics, including total coverage, proportion of deeply covered targets and initial mapping reads, indicated no difference between schizophrenic patients and healthy controls (Table 2).

Importantly, we found that schizophrenic patients enriched significantly more nonsynonymous and coding variants than controls (Table 2, *P*<0.001). Allele counts between schizophrenic cases and healthy subjects did not show significant difference after adjustment (Supplementary Information, section 4). We performed two series of gene-based tests: a one-sided burden test of an increased rare allele rate in cases (https://atgu.mgh.harvard.edu/plinkseq/) and the CALPHA test.^43^ Both tests indicated that gene NRK (Nik Related Kinase, NM_198465, chrX: 105132399...105199499) had increased rare allele rate in cases (*P*⩽1.0 × 10^−6^). However, even according to the most stringent Bofferroni correction (0.05/20000=2.5 × 10^−6^, 20 000 are the total gene number; the results of *NRK* gene still present significant statistical difference.^44^ Nine rare variants within NRK present in 13 schizophrenic patients, but not in healthy controls (Supplementary Table 1).

*NRK* is a protein-coding gene, which is associated with hypermobility syndrome, hyperinsulinemic and hypoglycemia. GO annotations related to this gene include protein serine/threonine kinase activity and small GTPase regulator activity. Previous studies did not denote any association between *NRK* and schizophrenia; however, an important paralog of this gene is *TAOK2*, which is essential for dendrite morphogenesis and has been associated with autism spectrum disorder.^45^

**Figure 1:**
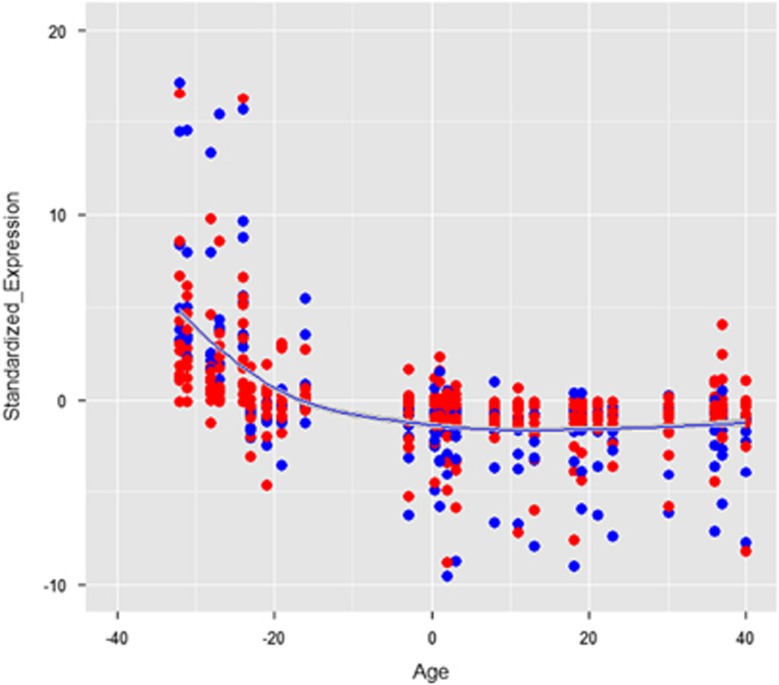
The developmental expression profile of 22 genes in the cell cycle pathway and 20 genes in the DNA repair pathway in hippocampus. Following colors for different Gene Ontology (GO) categories were used: red for DNA repair and blue for cell cycle, and a smooth curve with confidence interval (gray range). The expression profiles show high expression during the stage of brain development before birth, with a sharp decrease in expression after birth.

Next, we adopted an alternative strategy in which we studied RDVs. Previous reports have shown that RDVs have a higher likelihood of having a role in schizophrenia (Supplementary Information, section 5). After applying several filters (Supplementary Figure 1), 2895 mutations within 2442 genes in schizophrenic patients and 4484 mutations within 3481 genes in healthy controls were investigated with GO enrichment at the gene-based level using GeneMANIA. There were 505 and 952 pathway (or GO) terms enriched in cases and controls separately, but 56 RDVs in 42 genes within two pathways of DNA repair (GO: 0006281, 31 RDVs in 20 genes) and cell cycle (GO: 0007049, 25 RDVs in 22 genes) were present only in 39 schizophrenic patients (Supplementary Table 2 and Table 3), which also indicated a significant *q-*value. There are 60 control-only categories that were not statistical significant. In the present study, we focused on pathways damaged in cases only. We identified the developmental expression profile of 22 genes in cell cycle pathway and 20 genes in DNA repair pathway (Supplementary Information, section 6), which show high expression during the stage of brain development before birth, with a sharp decrease in expression after birth in hippocampus. The expression profile obtained by WGCNA is presented in Figure 1.

### Clinical characterization and hippocampal characterization in schizophrenic patients with or without RDVs

#### Clinical characterization

Comparisons here focus on schizophrenic and healthy subjects as well as on the two genetic-based subgroups with schizophrenia, that is, patients with RDVs and those without RDVs. There were no significant differences in age and sex among patients with RDVs, patients without RDVs and healthy controls (Table 4); however, the education years in patients without RDVs are significantly lower than those in patients with RDVs and controls. In addition, there were no significant differences in the duration of illnesses and severity of clinical symptoms between the two genetic-based patient subgroups.

#### rsFC of hippocampus

**Functional connectivity of bilateral hippocampus**

**Figure 2:**
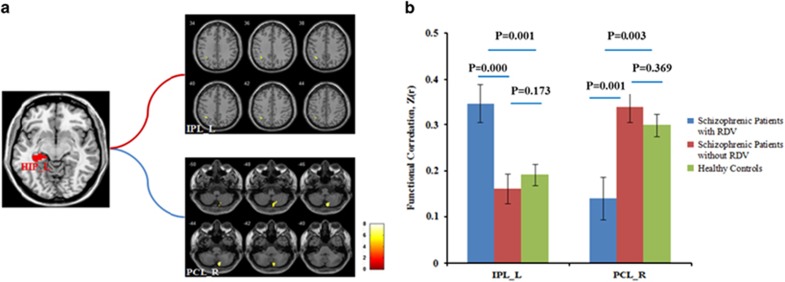
Aberrant resting-state functional connectivity (rsFC) of the left hippocampus among three groups. (**a**) Left inferior parietal cortex and right cerebellum posterior lobe showed significantly different rsFC with left hippocampus among three groups. The statistical significance threshold was set at *P*<0.05 and corrected for multiple comparisons based on Monte Carlo simulations, for main effect for diagnosis of functional connectivity (FC) of left hippocampus. (**b**) ANCOVA with sex, age, education and the hippocampal volume as covariances. rsFC between left hippocampus and left inferior parietal cortex is increased, whereas rsFC between left hippocampus and cerebellum is decreased only in schizophrenic patients with rare damage variants (RDVs). The statistical significance threshold was set at *P*<0.05, *post hoc* test by least significant difference. ANCOVA, analysis of covariance; HIP, hippocampus; IPL, inferior parietal cortex; L, left; PCL, cerebellum posterior lobe; R, right. Notes: s.e. for all figures.

Using analysis of covariance with sex, age, education and the hippocampal volume as covariances, we found that left hippocampus showed significant difference in rsFC with left inferior parietal cortex (Montreal Neurological Institute (MNI) atlas coordinates: *x*=−36, *y*=−56, *z*=43; voxels=42), and the right cerebellar posterior lobe (MNI coordinate: *x*=9, *y*=−75, *z*=−45; voxels=40) between the three groups at *P*<0.05 (corrected for multiple comparisons based on Monte Carlo simulations; Figure 2a). *Post hoc* test indicated that, compared with healthy controls and patients without RDVs, patients with RDVs demonstrated increased rsFC between left hippocampus and left inferior parietal cortex, as well as decreased rsFC between left hippocampus and right cerebellum posterior lobe. In contrast, there was no significantly different rsFC between healthy controls and patients without RDVs (Figure 2b). Unlike in the left hippocampus, there were no significant rsFC differences in the right hippocampus with other brain regions among the three groups.

**rsFC of the six hippocampal subregions**

Analysis of covariance showed significant differences in rsFC between the left dentate gyrus (DG) and left inferior parietal cortex (MNI coordinate: *x*=−36, *y*=−54, *z*=42; voxels=77), between right DG and left inferior parietal cortex (MNI coordinate: *x*=−48, *y*=−54, *z*=48; voxels=46) as well as posterior cingulate cortex (MNI coordinate: *x*=−6, *y*=−33, *z*=30; voxels=33), between left CA and right calcarine (MNI coordinate: *x*=24, *y*=−93, *z*=0; voxels=68), between right CA and right calcarine (MNI coordinate: *x*=21, *y*=−93, *z*=3; voxels=68) as well as left fusiform (MNI coordinate: *x*=−27, *y*=−54, *z*=−3; voxels=32), between left SC and orbital medial frontal cortex (MNI coordinate: *x*=3, *y*=30, *z*=−12; voxels=37) among the three groups at *P*<0.05 (corrected for multiple comparisons based on Monte Carlo simulations).

**Figure 3:**
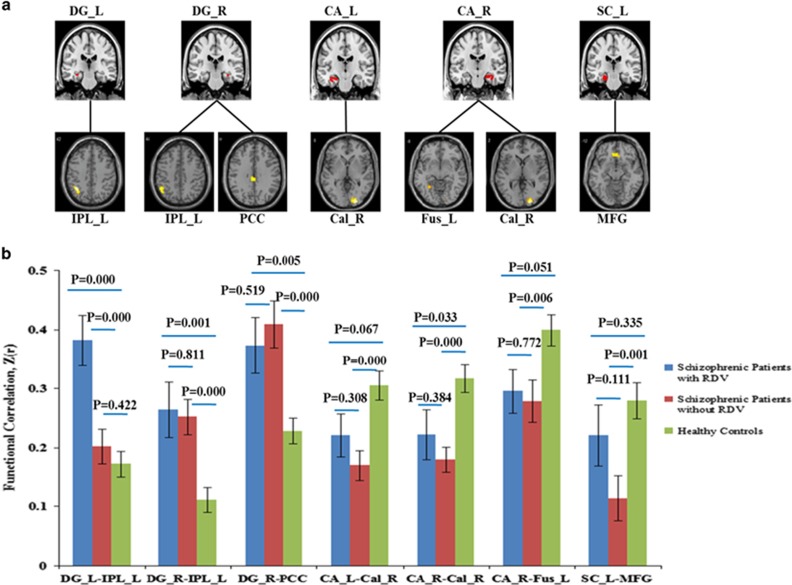
Aberrant resting-state functional connectivity (rsFC) of hippocampal subregions. (**a**) The brain map of aberrant rsFC with hippocampal subregions among three groups. The statistical significance threshold was set at *P*<0.05, corrected for multiple comparisons based on Monte Carlo simulations, for main effect for diagnosis of functional connectivity (FC) of the different hippocampal subregions. (**b**) rsFC between left DG and left IPL increased only in schizophrenic patients with rare damage variants, whereas decreased rsFC of left SC with MFG was only found in schizophrenic patients without rare damage variants. The statistical significance threshold was set at *P*<0.05, *post hoc* test by least significant difference. CA, cornuammonis; Cal, calcarine; DG, dentate gyrus; Fus, fusiform; IPL, inferior parietal cortex; L, left; MFG, medial frontal cortex; PCC, posterior cingulate cortex; R, right; RDV, rare damaging variant; SC, subicular complex. Notes: s.e. for all figures.

*Post hoc* tests indicated that increased rsFC between left DG and left inferior parietal cortex was only found in patients with RDVs, whereas increased rsFC between right DG and left inferior parietal cortex as well as posterior cingulum was detected in both patients group. rsFC of CA was significantly decreased in patients without RDVs and a trend decreased in patients with RDVs. Decreased rsFC of left SC and orbital medial frontal cortex was found only in patients without RDVs (Table 3, Figure 3).

### Relationships between hippocampal rsFC and memory

**Figure 4:**
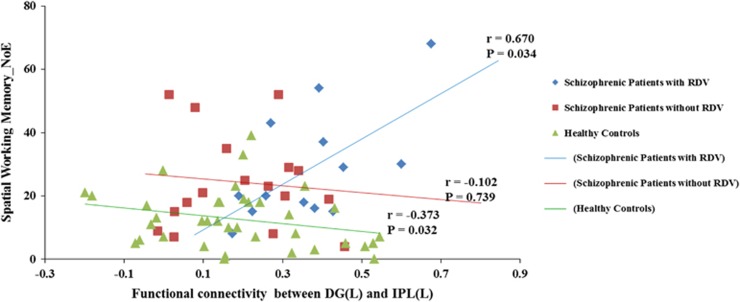
Relationships between resting-state functional connectivity (rsFC) of hippocampus and spatial working memory. Increased rsFC between left dentate gyrus and left inferior parietal cortex was positively related to number of errors of spatial working memory in schizophrenic patients with rare damaging variants and negatively related to number of errors of spatial working memory in healthy controls, uncorrected *P*<0.05. The difference in the degree of correlation was statistically significant between schizophrenic patients with RDVs and healthy controls (*Z*=3.331, *P*=8.65 × 10^−^^4^) after the Bonferroni correction, and a trend toward significance between patients with RDVs and patients without RDVs (Z=2.171, *P*=0.030 before Bonferroni correction), but not significant between patients without RDVs and healthy controls (*Z*=0.884, *P*=0.377). DG, dentate gyrus; IPL, inferior parietal cortex; NoE, number of error; L, left; RDV, rare damaging variant. Note: partial correlation analysis was used to analyze the relationship between rsFC of hippocampus and spatial working memory with age, sex and educations and structural hippocampal volume as covariates.

Compared with healthy controls, both patient groups showed significant impairments in SWM as well as in immediate and delayed logical memory (Supplementary Figure 4). No significant differences in these measures were observed between the two genetic-based subgroups in schizophrenic patients, that is, with and without RDVs (Supplementary Figure 4). Despite the lack of memory-related differences between the two genetic subgroups, we nevertheless observed different correlation patterns between rsFC and cognitive deficits. Errors in SWM were positively correlated to rsFC between left DG and left inferior parietal cortex in schizophrenic patients with RDVs (*r*=0.670, uncorrected *P*=0.034), whereas they were negatively correlated to rsFC between left DG and left inferior parietal cortex in healthy controls (*r*=−0.373, uncorrected *P*=0.032; Figure 4). However, after correction for multiple tests, none of correlations above remains significant statistically, probably because of the small sample size. No correlation was observed in schizophrenic patients without RDV (*r*=−0.102, uncorrected *P*=0.739).

We then compared the correlation coefficient between each of the two groups by using *Z*-test. We found that the difference in correlations was statistically significant between schizophrenic patients with RDVs and healthy controls (*Z*=3.331, *P*=8.65 × 10^−4^), as well as between patients with RDVs and those without RDVs (*Z*=2.171, *P*=0.030) after Bonferroni correction. No significant difference was found between patients without RDVs and healthy controls (*Z*=0.884, *P*=0.377).

## Discussion

Investigating a unique large sample of first-episode treatment-naive schizophrenic patients, we first identified that 56 RDVs in 42 genes within two pathways implicated in DNA repair and cell cycle were present only in a subgroup including 39 schizophrenic patients, whereas the remaining schizophrenic patients (that is, those without RDVs) as well as healthy subjects did not show these RDVs. Further analysis revealed that these genes are highly expressed during the stage of brain development before birth, with a sharp decreased expression after birth in specifically the hippocampus. Second, we identified a unique hippocampus rsFC pattern in schizophrenic patients with RDVs, that is, increased rsFC between left hippocampus (especially for left dentate gyrus) and left inferior parietal cortex, as well as decreased rsFC between the left hippocampus and cerebellum. Subsequently, we found a tentative significant correlation of altered hippocampal rsFC with spatial memory deficits in schizophrenic patients with RDVs.

Schizophrenic patients harbor more than threefolds rare DNA mutations, especially in those with early onset of illness.^14^ More *de novo* mutations were found recently at genomic hotspots, including chromosomes 1q21.1, 15q13.3, 16p13.1 and 22q11.2.^46^ Studies in larger sample sets demonstrated a polygenic burden that increases the risk for schizophrenia, those genes primarily comprising many ultrarare nonsense mutations distributed across many genes, which mainly involved in neurodevelopment pathways.^13^ Considering the low frequency and larger effect sizes of rare DNA mutations in current studies, we focused here on the case-unique RDVs, and found that these variants enriched in two pathways, for example, cell cycle regulation and DNA repair. Our finding in current study supported that disturbances of cell cycle regulation and DNA repair in post-mitotic neurons have been implicated in development of psychotic disorders.^25, 47, 48, 49^

Importantly, most of the genes enrichment in these two pathways was detected to be highly expressed in the hippocampus in the fetal stage, with the expression level sharply decreasing after birth. On the basis of this finding, we could hypothesize that individuals possessing mutations in these two particular pathways may be more prone to develop schizophrenia later on because of the critical gene expressions during their fetal development. More interestingly, such hypothesis genetically specifies and conforms well with the neurodevelopmental model that emphasizes insults as early as late-first or early-second trimester as central for pathological activation of neural circuits in schizophrenia^14, 50, 51^

Our finding of the RDVs in these two pathways hints upon alterations in cell cycle regulation and the DNA repair in schizophrenic patients during embryogenesis, although the underlying mechanisms are still in explicit. Study from Katsel *et al.* suggested abnormal patterns of cell cycle gene and protein expression in schizophrenia, which may contribute to the oligodendroglial deficits observed in schizophrenia.^52^ This could lead to changes in brain development and make the brain more susceptible to environmental risk factors, with downstream effects on neural progenitor proliferation and differentiation.^53^

In the present study, we detected aberrant functional connectivity of left hippocampus with inferior parietal lobe, posterior cingulate cortex, visual cortex and medial frontal gyrus in schizophrenic patients with RDVs. In contrast, schizophrenic patients without RDVs did not show such pattern. This suggests that the aberrant resting-state functional connections of left hippocampus in schizophrenia might likely be of neurodevelopmental origin (rather than of neurodegenerative origin or affected by antipsychotics with the latter being excluded here anyway because of the fact that our sample of treatment-naive). Most interesting, we found that increased functional connectivity of left hippocampus (especially for the dentate gyrus) with left inferior parietal cortex and decreased FC between left hippocampus and cerebellum were only found in schizophrenic patients with RDVs. This further underlines the neurodevelopmental impact of RDV on phenotypic makers like the left hippocampal rsFC and its high relevance for schizophrenia. The exact mechanisms mediating the transition from early prenatal RDVs' occurrence to hippocampal rsFC abnormalities during the later outbreak of schizophrenia remain unclear though.

Increased functional connectivity between left dentate gyrus and left inferior parietal cortex was correlated with more errors of SWM in schizophrenic patients with RDVs, whereas no such correlation was found in schizophrenic patients without RDVs (and opposite, that is, negative) and in healthy subjects. The dentate gyrus has been regarded as one of a few brain structures owning high rates of adult neurogenesis and has a critical role in resolving new memories and spatial memory.^54, 55, 56^ A recent study demonstrated that the inferior parietal cortex is involved in spatial perception and spatial orientation in particular and spatial functions in general (the ‘where').^52^ In addition, the inferior parietal cortex is also the target of output from hippocampus.^57^ The findings in the present study thus provided direct evidence that aberrant increased FC of left dentate gyrus with inferior parietal cortex might contribute to the severity of SWM deficits in schizophrenic patients with RDVs. Above all, our results provide a link among RDVs, dentate gyrus dysfunctional resting-state connectivity and SWM deficits in a genetic-based neurodevelopmental subgroup of patients with schizophrenia.

However, several limitations must be born in mind when interpreting our results. First, the sample size of the present study is relatively small for genetic study, which might be lack of the power to detect significant association signal between cases and controls. However, we adopt an alternative strategy for filtering rare variants and then enrich to find two special pathways. Second, not all the subjects underwent the memory test in both the two-patient groups, which could have affected the results of correlation analysis between imaging and cognitive data. Fortunately, there were no significant differences in clinical profiles between patients with RDVs who underwent cognitive tests and those who did not within both groups. Third, considering the small sample size of patients who underwent the cognitive tasks, the correlation analysis between neuroimaging and neurocognition remained uncorrected for multiple comparisons, which might lead to false-positive results.

In sum, to our knowledge, our findings demonstrate for the first time the presence of genes implicated in DNA repair and cell cycle pathways and related specifically to hippocampal development in a subgroup of first-episode treatment-naive schizophrenic patients. Importantly, the presence of these genes directly had an impact on or modulated phenotypic expression as hippocampal rsFC and related spatial memory function in this subgroup. Hence, our findings bridge the gap from genes regulating hippocampal development before birth to corresponding phenotypical markers like rsFC of hippocampus and associated cognitive, that is, SWM function at the outbreak of first-episode schizophrenia.

## Figures and Tables

**Table 1:** Statistic of demographic data for all samples

	*Case (*N=*94)*	*Control (*N=*134)*	P*-value*
Gender (male/female)	46/48	71/63	0.64
Age (mean±s.d)	23.94±6.92	24.39±10.95	0.37
Education (mean±s.d)	12.79±3.15	13.50±2.89	0.07

**Table 2:** Summary for sequencing quality

	*Total (*N=*228)*	*Case (*N=*94)*	*Control (*N=*134)*	P*-value*
Total reads	75 918 287	76 078 777	75 805 704	0.45
Total yield (bp)	7 667 746 978	7 683 956 451	7 656 376 154	0.45
Initial mappable reads (%)	75 731 958 (99.75%)	75 897 153 (99.76%)	75 616 076 (99.75%)	0.44
1 ×, Target genotypes (%)	58 570 040 (94.34%)	58 500 027 (94.23%)	58 619 153 (94.42%)	0.08
10 ×, Target genotypes (%)	55 003 180 (88.60%)	54 965 986 (88.53%)	55 029 271 (88.64%)	0.33
Mean target depth	44.95	46.54	43.84	**0.009**
SNPs	74 413	74501	74351	0.25
Coding SNPs	20 229	20157	20280	**<0.001**
Synonymous SNPs	10 491	10457	10516	**0.001**
Nonsynonymous SNPs	9220	9185	9245	**<0.001**
Indels	7645	7664	7632	0.19
Coding indels	383	378	386	**<0.001**

Abbreviation: SNP, single-nucleotide polymorphism.

Bold values show significant statistic difference.

**Table 3:** Aberrant functional connectivity of hippocampus and hippocampal subregions in patients group

	*Brain region*	*Peak MNI (*x y z*)*	*Peak* Z*-score*	*Cluster size*
Hippocampus_L	Inferior parietal lobule_L	−36 −56 43	6.8463	42
	Cerebellar posterior lobe	9 −75 −45	7.3691	40
				
*Hippocampal subregions*				
DG_L	Inferior parietal lobule_L	−36 −54 42	10.7382	77
DG_R	Inferior parietal lobule_L	−48 −54 48	8.2654	46
	Posterior cingulate cortex	−6 −33 30	7.0922	33
CA_L	Calcarine_R	24 −93 0	11.1746	68
CA_R	Calcarine_R	21 −93 3	13.0346	97
	Fusiform extent to lingual_L	−27 −54 −3	9.205	32
SUB_L	Medial frontal cortex	3 30 −12	7.1874	37

Abbreviations: CA, cornuammonis; DG, dentate gyrus; L, left; MNI, Montreal Neurological Institute; SC, subicular complex; R, right.

Note: The statistical significance threshold for main effect for diagnosis of functional connectivity of each hippocampal subregions was set at *P*<0.05, corrected for multiple comparisons based on Monte Carlo simulations.

**Table 4:** Sample summary and test scores for imaging

*Category (mean±s.d.)*	*Cases with RDVs (*n=*26)*	*Cases without RDVs (*n=*48)*	*Control (*n=*74)*	*df*	F/t/λ*/value*	P-*value*
Age (years)	25.69±7.2	23.00±6.92	23.23±6.52	2	1.54	0.22
Gender (male/female)	13/13	24/24	41/33	2	0.43	0.81
Years of education	13.42±3.19	11.90±3.08	13.44±3.24	2	3.72	0.03
Duration of disease	3.5	2		72	0.57	0.57
PANSS total	87.08±17.16	88.06±12.55		71	−0.28	0.78
PANSS positive	23.65±8.27	24.34±5.55		37.72	−0.38	0.71
PANSS negative	18.69±8.50	18.87±6.75		42.68	−0.09	0.93
PANSS general psychopathology	44.73±8.84	44.85±7.47		71	−0.06	0.95
Logical memory immediate	6.16±3.31	7.84±4.62	12.94±3.89	2	45.47	<0.001
Logical memory delay	4.09±3.19	5.65±4.39	11.02±4.12	2	44.51	<0.001
Hippocampus L milliliter	2534.70±291.31	2512.42±239.22	2722.55±301.57	2	7.42	0.001
Hippocampus R milliliter	2573.12±305.71	2559.78±262.83	2741.44±289.61	2	5.63	0.004

Abbreviations: L, left; PANSS, Positive and Negative Syndrome Scale; R, right; RDV, rare damage variant.

Note: values are mean (s.d.).
