# Supplementary material for: Rare damaging variants in DNA repair and cell cycle pathways are associated with hippocampal and cognitive dysfunction: a combined genetic imaging study in first-episode treatment-naive patients with schizophrenia
Source: Transl Psychiatry. 2017 Feb 14;7(2):e1028–. doi: 10.1038/tp.2016.291 (PMC5438026; doi:10.1038/tp.2016.291)
Supplement: Supplementary Information [file tp2016291x1.docx]

**Supplementary methods:**

1. Samples collection

2. Exome sequencing and variants calling

3. Quality control

4. Single-site and gene-based association analysis

5. Alternative strategy for filtering rare variants

6. Weighted gene co-expression network analysis (WGCNA)

7. Samples for imaging

8. Magnetic resonance imaging (MRI) data acquisition

9. Processing and statistical analysis of the imaging data

10. Supplementary Figures

1. **Samples Collection:**

All sequenced samples are Han Chinese from Sichuan province of China, consisting of 97 schizophrenia patients and 137 normal controls. All patients with schizophrenia were recruited at the Mental Health Centre of the West China Hospital, Sichuan University, People’s Republic of China and the diagnoses of schizophrenia were confirmed by an attending psychiatrist and a trained psychiatrist. Case inclusion criteria: ①Fulfilling the diagnostic criteria for schizophrenia as specified in the structured clinical interview for DSM-IV (Diagnostic and Statistical Manual of Mental Disorders, fourth edition)-PatientVersion (SCID-P)[^1^](#_ENREF_1); ② First-episode patients. Case exclusion criteria: Other SCID-P diagnosis or a neurological or medical condition; ②Severe physical illness; ③History of consciousness disturbance after traumatic brain injury. All patients have been followed up for at least 6 months in order to confirm the diagnosis of schizophrenia. 140 healthy volunteers were recruited from the community. Control inclusion criteria:①Screen for a lifetime absence of psychiatric illnesses using the DSM-IV-NonPatient Edition (SCID-NP).②Excluded significant physical illnesses, pregnancies, or any psychiatric disorders. Ethics Committee of the West China Hospital of Sichuan University approved all procedures. All next of kin, carertakers or guardians consented on the behalf of participants to provided written informed consent for their participation. We used the following criteria to evaluate whether the participants had the capacity to consent: Firstly, patients have the ability to understand; Secondly, patients have the ability to know reason; Thirdly, patients have the ability to make rational decisions. If participants failed to fill out the consent form more than twice, their guardians were asked to fill out the consent on the behalf of patients.

1. **Exome sequencing and variants calling:**

Peripheral blood was collected from all participants, and genomic DNA was extracted according to a standard phenol–chloroform procedure. Exome sequencing was performed by Macrogen (http://www.macrogen.com/), a commercial service. DNAsample was prepared according to the Illumina Protocol. Targeted enrichment was performed with TruSeqExome Enrichment Kit optimized for Illumina sequencing. Briefly, genomic DNA was sheared to 350-400 base pair using Illumina adapters. The fragment was end repaired and polyA was ligated to the 3'and 5' end and Illumina adapters. The size selected product was PCR amplified, and the final product was validated using the Agilent Bioanalyzer. Two steps of hybridization and wash were needed for construction. PCR was used to amplify the enriched DNA library for sequencing which produced 101bp end reads, approximately 6-10 billion base calls were generated for each sample. PCR was performed with the same PCR primer cocktail used in TruSeq DNA Sample Preparation. The Enrichment Kit is designed to cover 62 Mbexomic sequences. Pipeline of raw data processing and calling was present in **Supplementary Figure 1**.Quality control of raw data was conducted by FastQCsoftware ([http://www.bioinformatics.babraham.ac.uk/projects /fastqc/](http://www.bioinformatics.babraham.ac.uk/projects%20/fastqc/)).Reads were mapped to a custom hg19 build using Burrows-Wheeler Alignment tool (BWA)[^2^](#_ENREF_2). The duplicate reads were flagged using Picard-tools (<http://picard.sourceforge.net/>). GATK[^3^](#_ENREF_3) IndelRealigner module was used to realign reads around insertion/deletion (Indel) sites. Individual sequence data (in BAM format) was preprocessed as whole NGS community suggests which mainly include local Indel realignment, PCR duplicates removal and base quality recalibration. Read qualities were recalibrated using GATK Table Recalibration. GATK unified Genotyper module was then used to call variants (both SNVs and Indels) from multiple samples simultaneously, which create a single Variant Call Format (VCF) file. Raw read data were visualized using the Integrative Genome Viewer (IGV)[^4^](#_ENREF_4).

1. **Quality control:**

Individual level QC

Individual level quality control was conducted on raw and clean variant to make sure avoiding false positive variants. A suite of per-individual metrics, which included the total number of alternate alleles, dbSNP coverage (build137), and Transition/Transversion (Ti/Tv) ratio, and variant quality recalibration (VQSR) were calculated. From available exome data, we extracted common variants and estimated per-individual heterozygosity (~inbreeding), pairwise relatedness, and sex-check using PLINK[^5^](#_ENREF_5). We also use EIGENSOFT[^6^](#_ENREF_6) doing population stratification analysis.

After calculating all of metrics above, we remove 6 outliers (N_case_=3 and N_control_=3), all of results show in **Supplementary Figure2**. Using verifyBamID (<http://genome.sph.umich.edu/wiki/VerifyBamID>), we estimated that samples of 6 outliers had above 20% contamination. High levels of contamination can reduce the accuracy of variant calls and lead to false positives calls, so we discard these samples for following analysis.

Variants QC

Variants quality control was conducted by in house software KGGSeq (<http://statgenpro.psychiatry.hku.hk/limx/kggseq/doc/UserManual.html>)[^7^](#_ENREF_7),which were carefully designed to filter and prioritize gene variants in exome sequencing of rare Mendelian and common complex disorder. Variants were kept if ①The minimum overall sequencing quality scores≧50 (--seq-qual 50) andthe minimum overall mapping quality score≧20 (--seq-mq 20);②The minimal genotyping quality per genotype≧30 (--gty-qual 30) and the minimal read depth per genotype≧30 (--gty-qual 30); ③The fraction of the reads carrying alternative allele≦5% at a reference-allele homozygous genotype (--gty-af-ref 0.05), the fraction of the reads carrying alternative allele≧25% at a heterozygous genotype (--gty-af-het 0.25), and the fraction of the reads carrying alternative allele≧75% at a alternative-allele homozygous genotype(--gty-af-alt 0.75);④Minimal observed number of non-missing genotypes in all samples as 50 (--min-obs 50); ⑤Variants in controls with the Hardy-Weinberg test p value≧0.00001 (--hwe-control 1.0E-5);⑥Variants with "FILTER"matching the VQSRlabels (--vcf-filter-inPASS, VQSRTrancheSNP90.00to93.00,VQSRTrancheSNP93.00to95.00,VQSRTrancheSNP95.00to97.00,VQSRTrancheSNP97.00to99.00).

1. **Single-site and gene-based association analysis:**

Methods implemented in PLINK/Seq (<http://atgu.mgh.harvard.edu/plinkseq/>) were employed for single site and gene based association analysis. Using a permutation scheme (--perm -1) to control for potential confounding effects, allele counts between cases and controls did not show very significant difference after adjustment. We evaluated evidence for association at the gene level using the basic burden test and the CALPHA test [^8^](#_ENREF_8). Both of these test indicated that gene *NRK* (Nik Related Kinase, NM_198465, chrX: 105132399...105199499) were detected for a very small P-values (p ≦1.0E-06).

1. **Alternative strategy for filtering rare variants**

Base on previous knowledge of highly genetic heterogeneity[^9^](#_ENREF_9) and the contribution of rare variants in schizophrenia[^10-12^](#_ENREF_10), we focus on the difference of very rare variants distribution between cases and controls. So we design a personality analysis pipeline. In current methods, we split the variants for case-unique and control-unique (**Supplementary Figure 1**) respectively. Variants were kept if ①Nonsynonymous; All mutations were annotation by KGGSeq, we ignored synonymous variants because nucleotide substitution of these kind variants does not change amino acid and we suppose theses mutations contributed little to the cause of schizophrenia.②Predict damaging; All of nonsynonymous variants that met any of the following criteriawere considered potentially damaging: frameshift, nonsense, stoploss, stopgain, splicing and missense mutation with Polyphen score≧0.90 and/or SIFT p≦0.05 and/orGrantham score≧100 and/or phyloP score≧2.0[^13-16^](#_ENREF_13). ③MAF(Minor Allele Frequency)≦0.1%;The reason why we focus on ultra rare variants is that shaun et al[^17^](#_ENREF_17) have proved these kinds of variants may contribute more to schizophrenia. In fact, after filtering by serious condition above, all of variants left proved to be very rare (MAF≦0.1%).④Variants within a gene express in braintissue. Brain expression data were extracted using existing database in the BrainSpan: Atlas of the Developing Human Brain (<http://hbatlas.org/>).

Variants filtering were conducted by KGGSeq[^7^](#_ENREF_7) mentioned above. After filtering, 2895 mutations within 2442 genes in cases and 4484 mutations within 3481 genes in controls were done Gene Ontology (GO) enrichment using GeneMANIA (<http://www.genemania.org/>).The results of enrichment indicated that two GO (GO: 0006281 and GO:0007049) only present in cases.

Candidate 52 variants within 41 genes were tested using standard Sanger sequencing on an ABI3730xl DNA Analyzer to validate presence of each mutation in the subjects, by designing custom primers (Sigma) based on ~500 bp of genomic sequence flanking each variant.

1. **Weighted gene co-expression network analysis (WGCNA)**

41 genes from two GO enrichments above were selected for weighted gene co-expression network analysis (WGCNA), which design based on the pair-wise correlations between gene expression profiles across different developmental stages to infer a gene co-expression network. Based on the network, clusters of highly correlate genes were detected. The WGCNA clusters the genes using a measure of topological overlap, based the correlation matrix raised to a power chosen to meet a scale-free topology criterion[^18^](#_ENREF_18), and all of which was done using the WGCNA R package[^19^](#_ENREF_19). All of Brain expression data from different points in life were acquired from a published study (The Human Brain Transcriptome; HBT)[^20^](#_ENREF_20).We use the standard parameters of the software package, with power set to 6 and a minimum module size of 10. In the resultant network, the expression levels of the genes in each module were represented by the first eigenvector of the correlation matrix between the genes (the module “epigene”). Plotting of epigene values was performed using the R package ggplot2[^21^](#_ENREF_21).

1. **Samples for imaging**

39 of 94 schizophrenic patients were detected with 52 variants in 41 genes in two pathways. So we divided cases into two groups (one group enrichment variants in two pathways, another group without such variants). Next, we checked hippocampus imaging between these three groups (cases with variants, cases without variants and controls).In total, 74 healthy controls, 48 patients without variants and 26 patients with variants underwent MRI scans.

1. **Magnetic resonance imaging (MRI) data acquisition**

Participants underwent MRI scans in the Department of Radiology at West China Hospital with a Signa 3.0-T scanner (GE Medical Systems, Milwaukee, WI) with an eight-channel phase array head coil. High-resolution T1 images were acquired by 3D spoiled gradient echo sequence (SPGR). The sequence used in this protocol was as follow: TR=8.5 ms; TE=3.93 ms; flip angle =12°; thickness of slice=1 mm; single shot; field of view (FOV) =24 cm× 24 cm; matrix =256 × 256; size of voxel=0.47×0.47×1 mm3; in total, 156 slices of axial images were collected from a brain. Resting-state functional MRI (fMRI) images were obtained using a gradient–echo echo-planar imaging (EPI) sequence. The subject was instructed to lie inside the scanner, remained relaxed with eyes closed when T_2_^*^-weighted fMRI images were acquired with a gradient-echo echo-planar imaging (EPI) sequence: repetition time/echo time = 2000/30 milliseconds; flip angle = 90°; slice thickness = 5 mm (no slice gap), 30 axial slices; 64×64 matrix size; field of view = 240×240 mm^2^; voxel size = 3.75×3.75×5 mm^3^. Each fMRI run contained 200 image volumes. During the rsfMRI scanning, Brainwave 2.0 software was used to monitor the head motion of the subject. The head translational movement and rotation were less than 1.5 mm and 1.5°, respectively.

1. **Processing of the data**

*Hippocampal delineation and analysis*

The hippocampi from each brain were traced using MultiTracer (<http://bishopw.loni.ucla.edu/MultiTracer/MultiTracer.html>)[^22^](#_ENREF_22).The hippocampi were manually traced in coronal brain slices from anterior to posterior contiguously using standard protocol by two trained investigators (ML L and HY), blind to all of the demographic variables. Anatomical landmarks were confirmed in all three orthogonalviewing planes, and contours were drawn onmagnified images (4x) to facilitate the accurate identification of neuroanatomic boundaries and faithful tracking of small-scalefeatures. The hippocampal borders were determined by the temporal horn, choroidal fissure, uncal and ambient cisterns, and the gray-white junction between the subiculum and parahippocampal gyrus (Supplementary Figure 3).Volumes were obtained from the hippocampal surface tracings for use as dependent measures in statistical analyses.

To quantify inter-rater reliability between two different investigators (ML L and H Y), the hippocampus on six brains werer andomly selected to traced and the volume of hippocampus were calculated. The intra-class correlation coefficient for hippocampal volume was 0.89. To establish intra-rater reliability, the hippocampusfrom6 randomly chosen brains repeatedly contoured, the raters were 0.91 and 0.90 for ML L and H Y, respectively.

The volume of bilateral hippocampal subregions including cornuammonis (CA)1-3, CA4-dentate gyrus (DG), subicular complex (SC) were auto calculated using SPM Anantomy Toolbox Version 2.0,from modulated gray matter volume map of every subject by Diffeomorphic Anatomical Registration Through Exponentiated Lie algebra (DARTEL) toolbox in Statistical Parametric Mapping (SPM) 8. The details are as follows: all structural MRI scans were realigned manually according to the anterior commissure-posterior commissure line and were segmented into probability maps of gray matter (GM), white matter (WM), and cerebrospinal fluid using the ‘newsegment’ routine implemented in SPM8. Flow fields and a series of template images were produced by running the ‘DARTEL (create templates)’ routine using imported versions of the GM and WM generated in the previous step. The flow fields as well as the final template images were used to generate smoothed (6 mm full-width at halfmaximum isotropic Gaussian kernel), Jacobian modulated, and spatially normalized GM in Montreal NeurologicalInstitute (MNI) space.

Covariance analysis (ANCOVA) by least significant difference post hoc test was used to analysis the volume of hippocampus (Left and Right, respectively) and hippocampus subregions among three groups with gray matter, sex, age and duration of education as covariates, and significant differences was set at p< 0.05.

*rsfMRI image processing*

rsfMRI image processing was carried out using Statistical Parametric Mapping (SPM8, http://www.fil.ion.ucl.ac.uk/spm) and Data Processing Assistant for Resting-State fMRI (DPARSF)[^23^](#_ENREF_23). The first 10 time points were removed to allow the fMRI signal to reach steady state. Raw rsfMRI images were first slice time corrected and realigned, and were subsequently unwarped to correct for susceptibility-by-movement interaction. Next, nuisance covariates including six motion parameters, cerebrospinal fluid and white matter signals were regressed out and each image was spatially normalized to the Montreal Neurological Institute (MNI) EPI template. Then, all images were linearly detrended and bandpass filtered (0.01–0.08 Hz) to eliminate the high-frequency physiological noise. Finally, and smoothed with a Gaussian kernel (full-width half maximum = 6mm).

*Hippocampus functional connectivity analyses: seed-to-voxel analysis*

Left and right hippocampus masks were derived from the Wake Forest University Pick atlas software, left and right hippocampal subregions including cornuammonis (CA)1-3, CA4-dentate gyrus (DG), subicular complex (SC)were auto-segment using SPMAnantomy Toolbox Version 2.0[^24^](#_ENREF_24)^,^ [^25^](#_ENREF_25) and applied to all preprocessed images after reslicing. The time courses averaged over all voxels of left /right hippocampus and six hippocampal subregions were extracted. Pearson correlation coefficients (r) between time courses of each amygdala and all other voxels were calculated and transformed to Fisher’s z scores to derive functional connectivity maps (FCM). One sample t-test was applied to test the connectivity of the right / left hippocampus within the healthy controls group (t>11 and cluster>500 voxels) (FCM of hippocampus in controls is shown in Supplementary Figure 4).

Statistical tests on the functional connectivity maps of hippocampus across groups were performed using analysis of covariance (ANCOVA) with sex, age and education years, and volume of hippocampus as covariates usingSPM8. The significant threshold were set at P<0.05, corrected for multiple comparisons based on Monte Carlo simulations. Subsequently, the mean Z value of each cluster with a significant FC difference was extracted and were compared by ANOVA followed by post hoc test in SPSS 18.0, across patients with RDVs, patients without RDVs and healthy controls((SPSS Inc., Chicago, IL), significant level of P values were set at less than 0.05.

*Relationships Between neuroimaging characters of hippocampus, clinical and neurocognitive variables*

Partial correlation analysis was used to analyze the relationship between the aberrant neuroimaging characters of hippocampus (volume and functional connectivity) and PANSS scores / logical memory/ spatial working memory with age, sex and educations as covariance. A significant statistical correlation was set at p<0.05, uncorrected.

1. **Supplementary Figures**


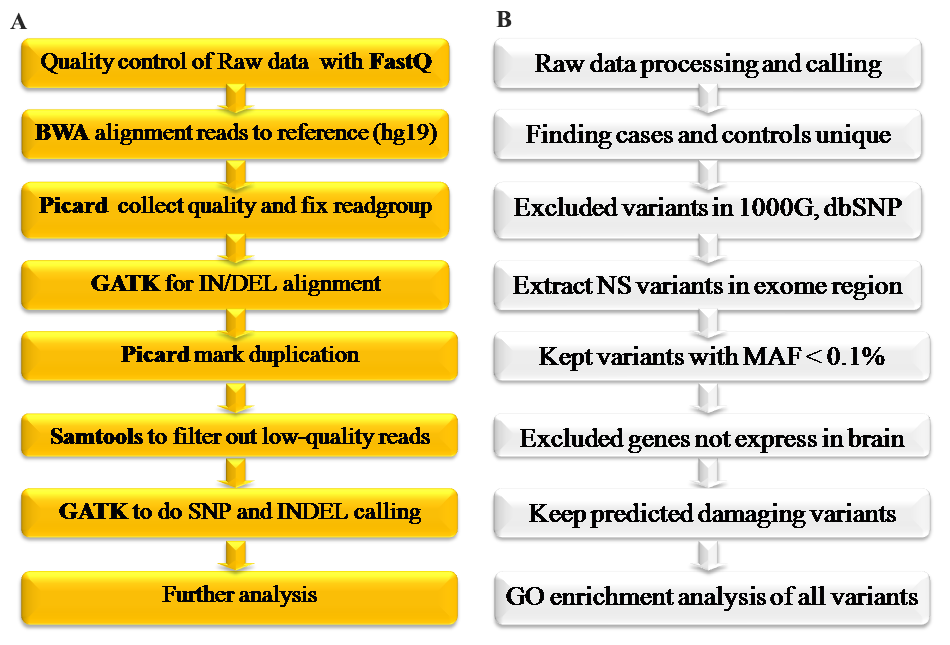
**Supplementary Figure 1| Pipeline of sequencing analysis and alternative strategy for filtering rare variants**

**A.** the pipeline describes the procedure for sequence data analysis. **B.** alternative strategy for filtering rare variants. All the variants after filtering must be nonsynonymous, very rare, predict damaging, and express in brain.


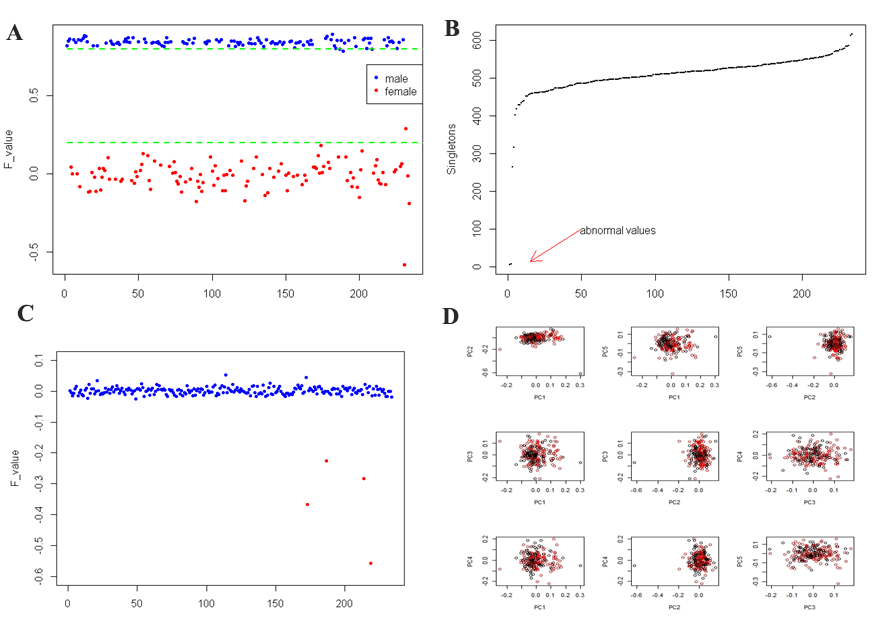


**Supplementary Figure 2 | Quality control of all samples before and after calling.**

**A.** we use option “Sex check” in Plink to flag individuals for whom does not match the estimated sex (consideration by genomic data). A male call is made if F value is more than 0.8 and a female is less than 0.2;

**B.** GATK software was employed for doing singletons analysis, four individual present abnormal values;

**C.** we use Plink to calculate inbreeding coefficients and results of F value in four individuals indicated strongly negative, which can reflect sample contamination et al.

**D.** Population stratification analysis was done use EIGENSOFT. The relationship between principal components (PC) was present. We mainly present 9 pairs of relationship between PC1 and PC5.


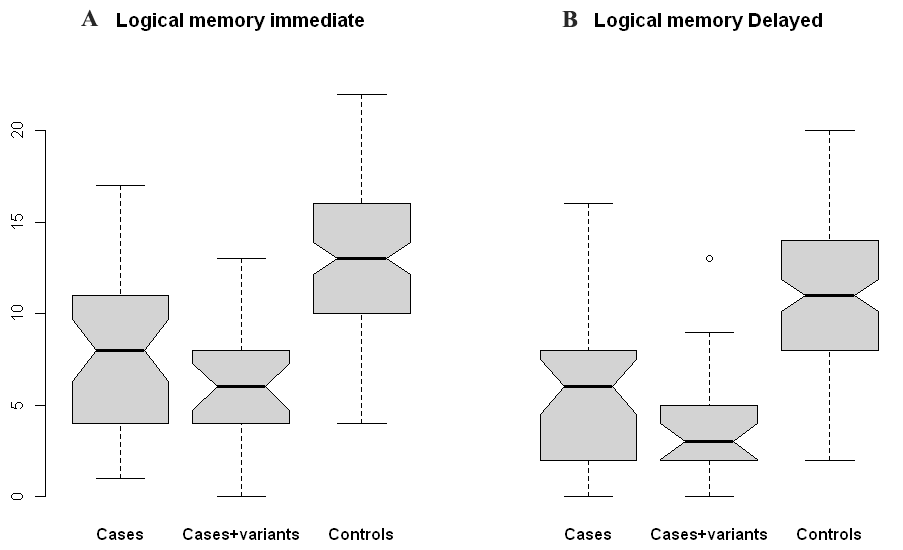


**Supplementary Figure 3| Comparison of neurocognitive score between three groups.**

Cases: Patients without two kind of GO enrichment.

Cases+variants: Patients with two kind of GO enrichment.

Controls: normal controls.

**A.**Neurocognitive score of immediatly logical memory were higher in controls, but no difference between cases with or without GO enrichment.

**B.** Neurocognitive score of delayed logical memory were higher in controls, but no difference between cases with or without GO enrichment.


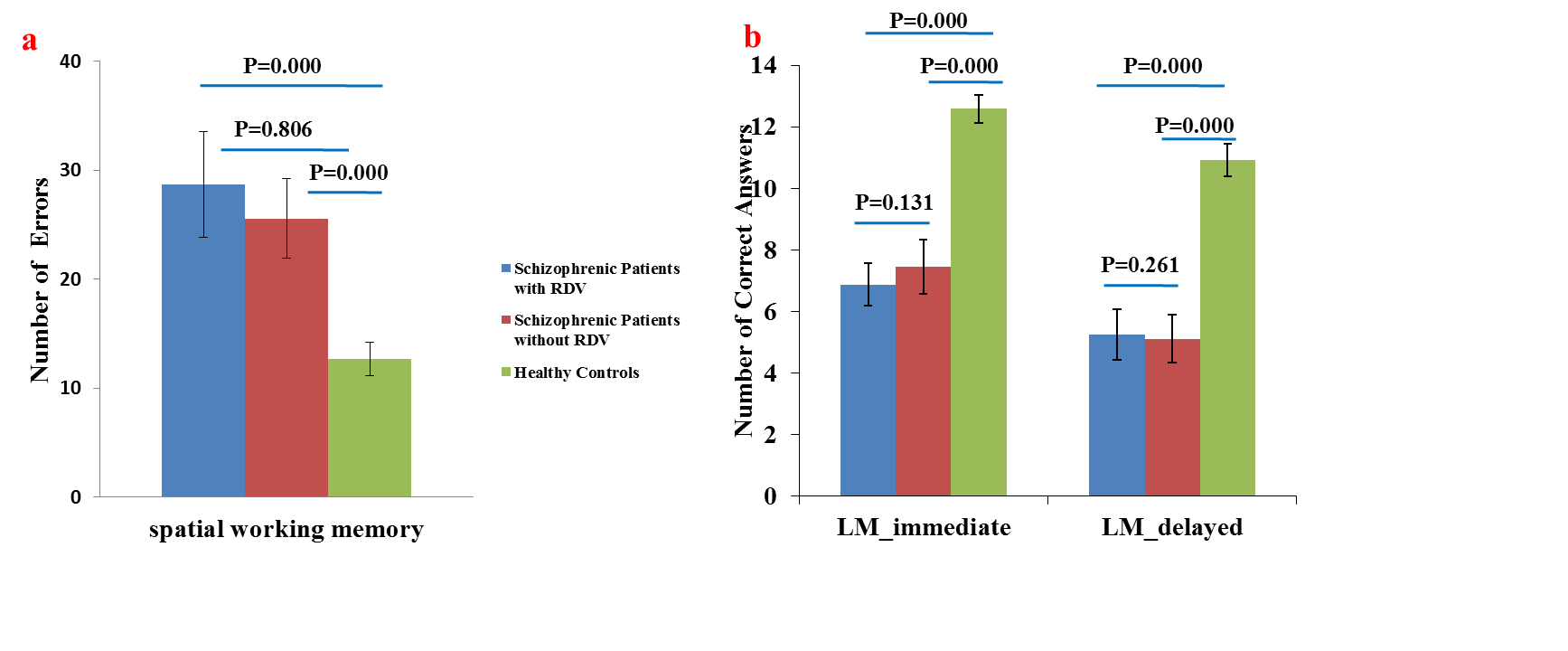


**Supplementary Figure 4| Memory differences among the three groups**

Relative to healthy controls, both cases groups showed impaired spatial working memory (Figure 2a) and immediate, delayed logical memory (Figure 2b). Analysis of covariance (ANCOVA) with age, sex and education as covariance, followed by post hoctest were applied to test the differences of memory among schizophrenic patients with rare damage variants, schizophrenic patients without rare damage variants and healthy controls.

Abbreviation：LM, logical memory; RDVs, rare damaging variants

Notes: Standard errorfor all figures


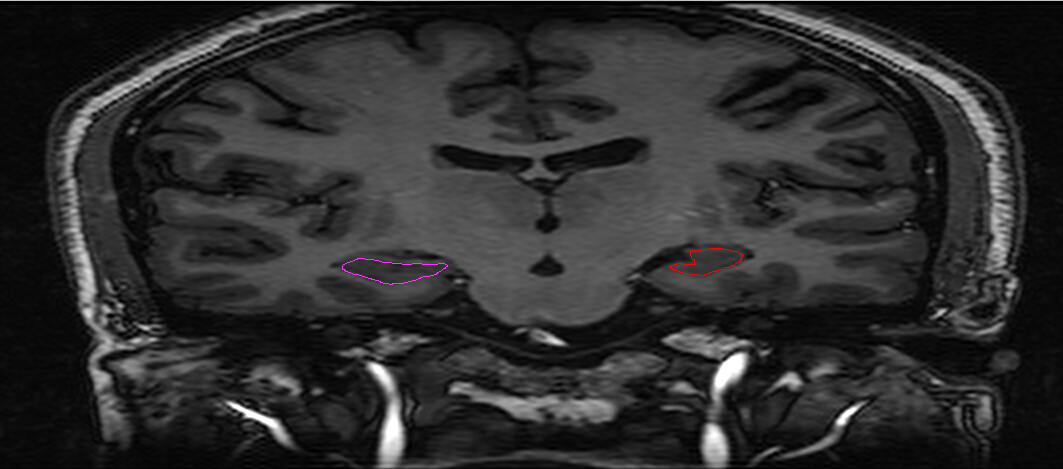


**Supplementary Figure 5| Manual traced of hippocampus**

The hippocampus were traced in high-resolution T1-weighted images using the software MultiTracer. The hippocampal borders were determined by the temporal horn, choroidal fissure, uncal and ambient cisterns, and the gray-white junction between the subiculum and parahippocampal gyrus.

**References**

1. First MB, Gibbon M. *User's guide for the Structured clinical interview for DSM-IV axis I disorders SCID-I: clinician version*. Amer Psychiatric Pub Incorporated, 1997.

2. Li H, Durbin R. Fast and accurate short read alignment with Burrows-Wheeler transform. *Bioinformatics* 2009; **25**(14)**:** 1754-1760.

3. McKenna A, Hanna M, Banks E, Sivachenko A, Cibulskis K, Kernytsky A *et al.* The Genome Analysis Toolkit: a MapReduce framework for analyzing next-generation DNA sequencing data. *Genome research* 2010; **20**(9)**:** 1297-1303.

4. Thorvaldsdóttir H, Robinson JT, Mesirov JP. Integrative Genomics Viewer (IGV): high-performance genomics data visualization and exploration. *Briefings in bioinformatics* 2013; **14**(2)**:** 178-192.

5. Purcell S, Neale B, Todd-Brown K, Thomas L, Ferreira MAR, Bender D *et al.* PLINK: a tool set for whole-genome association and population-based linkage analyses. *The American Journal of Human Genetics* 2007; **81**(3)**:** 559-575.

6. Price AL, Patterson NJ, Plenge RM, Weinblatt ME, Shadick NA, Reich D. Principal components analysis corrects for stratification in genome-wide association studies. *Nature genetics* 2006; **38**(8)**:** 904-909.

7. Li M-X, Gui H-S, Kwan JS, Bao S-Y, Sham PC. A comprehensive framework for prioritizing variants in exome sequencing studies of Mendelian diseases. *Nucleic acids research* 2012; **40**(7)**:** e53-e53.

8. Neale BM, Rivas MA, Voight BF, Altshuler D, Devlin B, Orho-Melander M *et al.* Testing for an unusual distribution of rare variants. *PLoS genetics* 2011; **7**(3)**:** e1001322.

9. McClellan J, King M-C. Genetic heterogeneity in human disease. *Cell* 2010; **141**(2)**:** 210-217.

10. Walsh T, McClellan JM, McCarthy SE, Addington AM, Pierce SB, Cooper GM *et al.* Rare structural variants disrupt multiple genes in neurodevelopmental pathways in schizophrenia. *Science* 2008; **320**(5875)**:** 539-543.

11. Xu B, Roos JL, Levy S, Van Rensburg E, Gogos JA, Karayiorgou M. Strong association of de novo copy number mutations with sporadic schizophrenia. *Nature genetics* 2008; **40**(7)**:** 880-885.

12. Stefansson H, Rujescu D, Cichon S, Pietiläinen OP, Ingason A, Steinberg S *et al.* Large recurrent microdeletions associated with schizophrenia. *Nature* 2008; **455**(7210)**:** 232-236.

13. Adzhubei IA, Schmidt S, Peshkin L, Ramensky VE, Gerasimova A, Bork P *et al.* A method and server for predicting damaging missense mutations. *Nature methods* 2010; **7**(4)**:** 248-249.

14. Kumar P, Henikoff S, Ng PC. Predicting the effects of coding non-synonymous variants on protein function using the SIFT algorithm. *Nature protocols* 2009; **4**(7)**:** 1073-1081.

15. Grantham R. Amino acid difference formula to help explain protein evolution. *Science* 1974; **185**(4154)**:** 862-864.

16. Pollard KS, Hubisz MJ, Rosenbloom KR, Siepel A. Detection of nonneutral substitution rates on mammalian phylogenies. *Genome research* 2010; **20**(1)**:** 110-121.

17. Purcell SM, Moran JL, Fromer M, Ruderfer D, Solovieff N, Roussos P *et al.* A polygenic burden of rare disruptive mutations in schizophrenia. *Nature* 2014.

18. Zhang B, Horvath S. A general framework for weighted gene co-expression network analysis. *Statistical applications in genetics and molecular biology* 2005; **4**(1)**:** 1128.

19. Langfelder P, Horvath S. WGCNA: an R package for weighted correlation network analysis. *BMC bioinformatics* 2008; **9**(1)**:** 559.

20. Kang HJ, Kawasawa YI, Cheng F, Zhu Y, Xu X, Li M *et al.* Spatio-temporal transcriptome of the human brain. *Nature* 2011; **478**(7370)**:** 483-489.

21. Wickham H. *ggplot2: elegant graphics for data analysis*. Springer, 2009.

22. Woods RP. Multitracer: a Java-based tool for anatomic delineation of grayscale volumetric images. *NeuroImage* 2003; **19**(4)**:** 1829-1834.

23. Chao-Gan Y, Yu-Feng Z. DPARSF: a MATLAB toolbox for “pipeline” data analysis of resting-state fMRI. *Frontiers in systems neuroscience* 2010; **4**.

24. Amunts K, Kedo O, Kindler M, Pieperhoff P, Mohlberg H, Shah NJ *et al.* Cytoarchitectonic mapping of the human amygdala, hippocampal region and entorhinal cortex: intersubject variability and probability maps. *Anatomy and embryology* 2005; **210**(5-6)**:** 343-352.

25. Eickhoff SB, Stephan KE, Mohlberg H, Grefkes C, Fink GR, Amunts K *et al.* A new SPM toolbox for combining probabilistic cytoarchitectonic maps and functional imaging data. *Neuroimage* 2005; **25**(4)**:** 1325-1335.
